# Ruptured Hepatocellular Carcinoma: Current Status of Research

**DOI:** 10.3389/fonc.2022.848903

**Published:** 2022-02-17

**Authors:** Feng Xia, Elijah Ndhlovu, Mingyu Zhang, Xiaoping Chen, Bixiang Zhang, Peng Zhu

**Affiliations:** ^1^ Department of Hepatic Surgery Center, Tongji Hospital of Tongji Medical College of Huazhong University of Science and Technology, Wuhan, China; ^2^ Department of Digestive Medicine. Tongji Hospital of Tongji Medical College in Huazhong University of Science and Technology, Wuhan, China

**Keywords:** hepatocellular carcinoma, spontaneous rupture, mechanism, diagnosis, treatment

## Abstract

**Background:**

Ruptured hepatocellular carcinoma (rHCC) is considered a rare and life-threatening manifestation; when it happens, it often requires acute and positive intervention. At present, the mechanism of rHCC development is gradually being understood while there are many kinds of rHCC treatment. From our clinical observation, the prognosis of rHCC patients is not as poor as it is currently believed. It may not be appropriate to include all patients with rHCC in T4.

**Main Body:**

The incidence of ruptured hepatocellular carcinoma is now rising. Especially in the Asian region, it can even reach 10% – 15%. The most common symptom of HCC rupture is abdominal pain, and there are now a variety of treatments for hepatocellular carcinoma rupture. With aggressive treatment, rHCC patients can also achieve a better prognosis. The patient’s condition varies on admission, so the treatment methods will also be different. It is critical to identify prognostic factors simultaneously, and rHCC can be effectively managed by focusing on important prognostic factors.

**Conclusion:**

A review was carried out to analyze diagnosis, mechanism, treatment, and prognostic risk factors on this disease condition during the current situation; it is hoped that it will provide better guidance for clinicians. Moreover, patients with rHCC were managed hierarchically to prolong their prognosis.

## Introduction

Hepatocellular carcinoma is one of the most common malignant tumors globally, with nearly 800,000 new deaths each year, and spontaneous rupture hemorrhage is one of the fatal complications (1–6). Many articles report an acute phase mortality rate of 25% – 75% due to HCC rupture. In recent years, the incidence of rHCC has been increasing, reaching as high as 10% – 15% in some parts of the Asian region, showing that rHCC is not rare, and we should pay more attention to it (7–9).

Ruptured HCC usually presents with abdominal symptoms (66% – 100%), and shock may occur in 33% of cases; 33% report abdominal distension, which at the same time leads to high mortality. Despite the continuous development of medical technology, the diagnosis and treatment of rHCC is still a great challenge (2, 8). Many studies have identified some of the risk factors for developing rupture in HCC, including tumor size > 5 cm, hypertension, cirrhosis, and vascular thrombosis.

In the previous literature, many investigators believe that (10) once HCC ruptures, the prognosis is very poor, and in TNM staging, patients with rHCC are directly included in T4. However, in recent years, the results of some studies and our own clinical experience suggest that rHCC patients can also achieve a better prognostic status through active treatment. By focusing on some prognostic factors, we can better manage patients with rHCC. It is not completely applicable to include all rHCC patients in T4, and some investigators have also proposed insights into improving staging (11, 12). Therefore, this review will discuss the risk factors, mechanisms, diagnosis and treatment, and prognostic factors of rHCC to referring clinicians.

## Clinical Manifestations and Diagnosis of Rupture and Hemorrhage of HCC

The clinical presentation of emergency patients with ruptured HCC is slightly different. RHCC patients are generally admitted to the emergency department with the following common clinical manifestations ① Peritonitis: Sudden severe epigastric pain, tenderness, rebound pain, and muscle tension ② Different degrees of hemorrhagic shock: dizziness, fainting, restlessness, palpitation, shortness of breath, thirst and fatigue, pulse speed, oliguria, “anemia appearance” and so on. ③ Hemoperitoneum: abdominal distension, extraction of non-coagulated blood upon diagnostic abdominal puncture, etc. ④ Liver cirrhosis and HCC: jaundice, liver palms, spider nevus, etc (3, 13, 14).

Many patients with ruptured HCC develop symptoms of liver dysfunction before admission. Therefore, liver and kidney function and coagulation function should be urgently examined on admission. The development of imaging technology has also improved diagnostic ability (15–18). Contrast-enhanced abdomen CT is also done to identify the tumor size and location. After admission, examination of blood routine, liver and kidney function, electrolytes, coagulation function, hepatitis serology, tumor markers, and other indicators are helpful to understand the basic status of patients.

US is a routine examination for evaluating ruptured HCC. It has been reported that the detection rate of US in the diagnosis of abdominal organs is more than 90% – 97% (19, 20) because it can show the size, number, location of the tumor, and the relationship with the surrounding blood vessels, and it can indicate whether the tumor has abdominal metastasis and whether the tumor compresses the vascular system. However, conventional US is ineffective in ruptured and hemorrhagic patients with smaller tumor diameters (21–23).

Computed tomography is a more useful technique for detecting rHCC. On unenhanced CT, the most obvious is the peritoneum and the surrounding hematoma. In addition, CT examination can help clinicians localize the source of bleeding, as hematomas with the greatest attenuation on CT scans are usually closest to the source of bleeding, while non-coagulated hematomas with less attenuation tend to be distant from the bleeding site. The size of HCC and the degree of extrahepatic protrusion are associated with subsequent rupture. In HCC rupture, the main CT findings are HCC beyond the Porto-hepatic margin and discontinuous or ruptured liver surfaces adjacent to or in contact with HCC. However, there are few sites of active bleeding (18, 24, 25).

Suppose patients who had previous hepatitis, cirrhosis, epigastric pain, and discomfort suddenly present with the shock of different degrees, or non-coagulated blood is extracted by an abdominal puncture. Combined with ultrasound and CT examination, it can be diagnosed as rupture and hemorrhage of primary HCC. However, the diagnosis should be differentiated from splenic rupture, ectopic pregnancy rupture, ovarian follicle or corpus luteum cyst rupture, and gastrointestinal perforation (26, 27).

## Mechanism of Rupture

Ruptured HCC is more common in nodular and massive HCC and rare in diffuse HCC. The mechanism of spontaneous rupture of HCC is still controversial. Some researchers believe it is related to the following factors: ① The size, location, and growth rate of the tumor. Studies have shown that tumor diameter more than 5-7 cm, tumor protruding more than 1 cm from the surface of the liver, tumor located in segments II, III, IV B, VI and are independent risk factors for spontaneous rupture and hemorrhage of HCC. The rapid growth of a single tumor and necrosis and local invasion inside the tumor may cause atresia of the branches of the hepatic vein, which further increases the pressure inside the tumor. The interaction of the two further increases intratumoral venous congestion. The above effects also aggravate elastin and collagen type 4 degradation and trigger vascular dysfunction, making the vessels stiff and brittle (3, 13, 28). ② Most patients with HCC have a long history of liver cirrhosis, and the synthetic reserve capacity of the liver is decreased. After rupture and hemorrhage, hypoperfusion injury aggravates the attack on the liver, which reduces the synthesis of coagulation factors, and leads to coagulation dysfunction (29, 30). ③ Positive HBsAg is an independent risk factor for rupture and hemorrhage of HCC, and the immunological destruction of tumor vasculature has gradually attracted people’s attention. The antigen-antibody complexes formed after the body gets infected with the hepatitis B virus are selectively deposited in the small artery walls and lead to the destruction of the normal structure of the vascular walls through local inflammatory reactions. At the same time, the function of macrophages in patients with HCC is damaged so that the antigen-antibody complexes of the hepatitis B virus cannot be effectively removed, and the collagen fibers in the small artery wall were sparse and broken. The vessel wall loses its supporting force, and its brittleness increases, so it is prone to rupture and bleeding under external force. Spontaneous rupture and hemorrhage of HCC are the results of the above factors. Notably, Jason C Smith et al. (31) have reported a case of HCC rupture after TACE. A full understanding of these factors is conducive to the early prevention and treatment of this emergency (32–35).

## Management of Ruptured HCC

Patients with impaired liver function tend to lapse into liver failure, which is the main cause of hypovolemic shock and hepatic hypoperfusion. Liver failure is also the main cause of death in these patients (36, 37). To prevent liver failure, the treatment principle of spontaneous rupture bleeding of HCC is to control bleeding timely and effectively while considering the comprehensive treatment of HCC. At present, the main treatment methods include conservative treatment, transcatheter arterial embolization (TAE), partial hepatectomy, TAE combined with partial hepatectomy, and other treatment methods. At the same time, the treatment sequence is also an important issue for some complex cases. TACE followed by elective surgery is currently considered the most effective treatment (4, 32, 33, 38). The results of some published treatment series of patients with rHCC, are summarized in **Table 1**.

**Table 1:** Main treatment methods and clinical results of ruptured hepatocellular carcinoma in a published series.

Study	Cases	N		Surgical-OS		TAE/TACE alone-OS	
		Surgical	TAE/TACE	Median time^a^	1-Year^b^	Median time^a^	1-Year^b^
Jixue Zou et al. (39)	70	31	39	8.0	54.8%	7.4	46.2%
Jae Hyun Kwon et al. (40)	89	89	0	43.6	88.8%	N/A	N/A
Kohei Shinmura et al. (41)	51	0	51	N/A	N/A	1.0	18%
E J Monroe et al. (42)	23	0	23	N/A	N/A	8.7	45%
Darren W.Chua et al. (43)	49	49	0	21.9	85.0%	N/A	N/A
Xinjian Xu et al. (44)	974	489	485	N/A	48.7%	N/A	47.6%
Hanteng Yang et al. (45)	69	28	41	13.1	56.3%	2.9	9.1%
Sada H et al. (46)	43	16	27	12.8	49.0%	2.5	8.3%
Liu Hai et al. (47)	135	135	0	60.0	88.8%	N/A	N/A
Jing Li et al. (48)	92	92	0	12.0	66.3%	N/A	N/A
Qian Zhu et al. (49)	106	106	0	10.0	37.7%	N/A	N/A
Hyung Soon Lee et al. (50)	105	44	61	48.0	88.7%	12.0	40.1%

OS, overall survival; ^a^ means median survival time,unit:month; ^b^ means one-year overall survival rate, unit:month; N/A, Not applicable. Some surgical patients received preoperative treatment.

### (1) Conservative Treatment

Conservative treatment mainly refers to absolute bed rest, avoiding severe cough, monitoring vital signs, active fluid infusion, anti-shock treatment, drug hemostasis, liver protection, anti-inflammatory treatment, and nutritional support. Because most of the HCC’s blood supply is from the hepatic artery, rHCC hemorrhage belongs to arterial hemorrhage, with the characteristics of acute onset, a large amount of bleeding, and critical condition. Conservative treatment has a high risk of rebleeding and mortality, and it is generally only applicable in the following conditions: ① Correcting the general state of the patient to prepare for operation; ② patients with stable vital signs, no active bleeding and/or small rupture; ③ patients with a poor general state and unable to tolerate TAE and operation; ④ patients with extensive metastasis of HCC.

Conservative treatment has a very poor prognosis in most studies (8, 51, 52), and conservative therapy alone is currently limited to patients in a moribund state (7, 51, 53, 54). Although conservative treatment has a certain hemostatic effect in a short period, it cannot achieve the fundamental control of rHCC, and the patients often die of hemorrhagic shock or liver failure again. It is not difficult to find that the overall prognosis of rHCC treated with conservative therapy alone is very poor. The main reasons are as follows: ① The probability of rebleeding in the process of conservative treatment is high, the patient’s condition is delayed, liver function deteriorates, and the prime opportunity of operation is lost; ② hemoperitoneum makes it easy for abdominal infection to occur, and provides a route for dissemination of the exfoliated cancer cells, increasing the probability of abdominal implantation and metastasis; ③ without resection of the lesion, the primary lesion can easily infiltrate and grow, which promotes the survival of intrahepatic and extrahepatic metastasis and intraperitoneal dissemination. Therefore, conservative treatment can improve the general situation of patients to a certain extent, but its effect is not ideal. So, it is recommended that simple conservative treatment is only suitable for dying patients with poor liver function and locally advanced tumors who cannot tolerate transcatheter arterial embolization (TAE) or surgery (39, 55–57).

### (2) Transcatheter Arterial Embolization (TAE)/Transcatheter Arterial Chemoembolization (TACE)

TAE/TACE has developed rapidly in recent years and is widely used to control acute bleeding. TAE/TACE uses the Seldinger technique to puncture and cannulate the femoral artery to confirm the tumor and bleeding and uses embolic agents such as iodized oil or gelatin sponge particles to block the target vessel. TAE/TACE is minimally invasive and has a high success rate for patients with rHCC, and can significantly improve the short-term prognosis (39, 40, 58–61). Therefore, emergency TAE/TACE therapy may be considered after diagnosis for patients with unstable vital signs and heavy bleeding volume to control bleeding effectively (39, 41, 42, 58, 62).

The main advantages of TAE are: ① the operation is performed under local anesthesia, which avoids the double impact of anesthesia and operation, and the patients have good tolerance; ② the success rate of hemostasis is 53% ∼ 100%, with definite effect and repeatable operation, which is conducive to the discovery of ectopic blood supply arteries. While super-selective vascular embolization can effectively stop bleeding, it also protects the function of normal liver tissue to the maximum extent, thus achieving precise treatment; ③ The arterial blood supply of HCC is about 95%, and TAE/TACE can block most of the blood supply of the tumor and inhibit swelling and tumor growth, allowing for easier secondary surgical treatment; ④ if the situation allows, fluorouracil, cisplatin, epirubicin, and other chemotherapy drugs can be injected to treat the primary disease; ⑤ It gives a clear picture of the tumor blood supply, the relationship between tumor and peripheral blood vessels, whether there is arteriovenous fistula and portal vein tumor thrombus, which serves as a reference for subsequent treatment (39, 41, 58, 60, 62–64).

According to some studies, the 30-day hospital mortality (0-37%) was significantly higher than traditional surgery (28% - 75%). Furthermore, the prognosis of patients with poor hemostatic effect or rupture of TAE is very poor, and the mean survival time of rHCC patients treated with TAE depended on the baseline liver function. Some studies showed that those with a bilirubin level below 50umol/L and those who presented with shock had a poor outcome. Therefore, we may believe that total bilirubin<50μmol/L and whether the patient presented with shock can be used as the critical value when considering TAE (65).

Studies have shown that the effect of combined surgery after TAE/TACE is better than TAE/TACE alone, chemotherapy, and conservative treatment. Therefore, it is recommended that after interventional hemostasis, patients with rHCC should strive for secondary surgical resection as soon as possible under the condition of gradual improvement of liver function and hemoglobin to improve the long-term prognosis. Reviewing the existing clinical studies, it is not difficult to find that the main advantages of interventional therapy compared with surgical resection are: ① super-selective embolization can ensure the normal blood supply of liver tissue to the greatest extent, and significantly reduce the incidence of various complications; ② It can safely and effectively achieve hemostasis, it can significantly prolong the survival time of patients allowing for improvement in the vital status of patients with a low tolerance to operation, and it is suitable for the vast majority of patients with rHCC because of small trauma and less pain (2, 8, 43, 52, 62).

For TAE with chemotherapy drugs, adjusting the dose of the embolic agents and chemotherapy drugs based on the tumor size, blood supply, liver function classification, and iodized oil precipitation state can reduce the risk of rebleeding and chemotherapy-induced liver injury. Through a meta-analysis, Xu et al. (44) stated that the results of TAE/TACE were comparable to emergency surgery in terms of successful hemostasis and 1-year survival rate. Jixue Zou et al. (39) also showed that conventional TACE therapy is safe and effective in treating spontaneous HCC rupture. Furthermore, this type of treatment has similar long-term survival rates to open surgery. Therefore, TAE/TACE may be recommended as the treatment of choice for rHCC.

#### Postembolization Syndrome

After TACE, most patients experience the post-embolization syndrome, which manifests as right upper abdominal discomfort, nausea, vomiting, etc. Therefore, patients and their families should be informed before TACE, and symptomatic treatment should be performed after symptoms occur. Apart from these, Alopecia, myelosuppression, leukopenia, and anemia may all occur following TACE (66). Some studies have shown that after transcatheter arterial chemoembolization (TACE), vascular endothelial growth factor (VEGF) was highly expressed in HCC tissue, promoting angiogenesis and increasing the chance of metastasis and recurrence. Therefore, it is suggested that radical surgery be performed as soon as possible after TACE. Due to the special location of the caudate lobe of the liver, the number and source of blood vessels vary a lot. Venous blood flows into the inferior vena cava through the short hepatic veins. At the same time, TAE treatment is difficult due to tumor collateral circulation, and the hemostatic effect is not accurate. TAE can lead to liver failure or even death when the tumor invades the portal vein with tumor thrombus formation (65–68).

### (3) Surgery

As a traditional treatment, surgical resection has been used for a long time to treat rHCC. According to the literature, the resection rate of rHCC is 12.5% ~ 59.3%, and the resection rate of R0 is 81% ~ 88%. A retrospective analysis of 89 rHCC patients found the overall survival rates at 1, 3, and 5 years were 87.1%, 65.4%, and 48.4% (69). It can be seen that surgical treatment of some patients with rHCC can achieve better therapeutic results. rHCC patients treated with liver resection have obvious survival benefits (26, 39, 41, 45, 46, 57, 59, 60, 70).

#### Emergency Hepatic Resection

Emergency resection of ruptured liver cancer was defined as surgery performed three days without other treatment. The main purpose of this surgical approach is to stop bleeding and prevent shock, but the ultimate goal is still radical resection. According to the literature, in-hospital mortality after emergency hepatectomy is high, usually 16.5 – 100% (47, 56, 71). Some patients were admitted with hemorrhagic shock when liver function and tumor stage were difficult to assess. Because many patients with rHCC have poor coagulation due to liver dysfunction, the shock state is further intensified. Since there is no specific guiding protocol to clarify the surgical indications for patients with rHCC, clinicians generally judge the operation time and mode by experience. The recent addition suggests that emergency hepatectomy can be performed in patients with better hepatic functional reserve on admission. However, emergency hepatectomy is performed for patients with unstable hemodynamic status or cirrhosis, albeit with a high mortality rate. Some studies also provided the general indications for liver resection; for example, Child grade A, without liver cirrhosis, platelet count greater than 100×10*9/L, and the ICG was ≤ 15% and good general condition (47, 48, 52, 54, 56, 62, 71–73).

Ruptured HCC can lead to peritoneal seeding (74, 75). Moreover, people support early emergency hepatectomy because early completion of hepatectomy and peritoneal lavage can reduce peritoneal dissemination. In a study, peritoneal metastasis was found in 10% of rHCC patients during the second stage hepatectomy, and about 1/3 of the patients had peritoneal dissemination during the follow-up. In another study, there was little peritoneal dissemination during the emergency operation, and only about 1/5 of the patients had peritoneal metastasis during the follow-up. However, the average in-hospital mortality of rHCC after emergency hepatectomy alone was 27%, which was significantly higher than that of secondary hepatectomy (52, 54, 62, 72).

#### Staged Hepatic Resection

Staged hepatectomy is defined as TACE and other treatments followed by hepatectomy after seven days (49). It is usually performed 14 to 42 days after an emergency admission when the patient’s vital signs have stabilized. There are many ways of hepatectomy, including anatomical hepatectomy, extensive hepatectomy, etc. The surgical resection method is usually selected at the surgeon’s discretion based on experience.

Intra-peritoneal lavage should be performed in all patients with staged hepatectomy and evacuation of the intra-abdominal hematoma. Previous studies have suggested that given the high mortality rate of emergency surgery, early secondary surgery after hemostasis can improve the preoperative status, reduce the perioperative risk and lower the abdominal dissemination rate than delayed surgery. Therefore, it is recommended to complete secondary surgery within eight days after hemostasis as soon as possible. Wu et al. (13) divided the patients after HCC rupture surgery into three groups, which were immediate resection of HCC rupture, resection within eight days of HCC rupture(staged early partial hepatectomy), and resection beyond eight days of HCC rupture(staged delayed partial hepatectomy). This study showed that staged early partial hepatectomy was better than staged delayed partial hepatectomy, with less peritoneal dissemination, better survival time, improved quality of life, and decreased hospital stay and cost.

TAE combined with secondary hepatectomy is an ideal treatment strategy for patients not suitable for emergency surgery (50). It is reported that compared with other treatment methods, the second stage partial hepatectomy after TACE can stop bleeding in time and has the advantages of less perioperative blood transfusion, less degree of liver function damage, fewer postoperative complications, greatly reduced in-hospital mortality, and prolonged survival. However, Liu et al. believed that although TACE/TAE has the above advantages, hepatic inflammation and adhesion after TACE is serious, increasing the difficulty of surgery, and postoperative complications such as bile leakage and bleeding are likely to occur. The timing of the second stage partial hepatectomy after TACE mainly depends on the tumor necrosis and shrinkage after TACE. Generally, the operation should be performed from one week to 6 months after TACE/TAE. If the interval is too long and the collateral circulation of the tumor is reconstructed, the tumor may grow again, leading to intrahepatic metastasis will occur, and loss of the chance for surgical resection of the tumor (47, 48, 52, 54, 56, 62, 71–73). In a multicenter analysis of staged partial hepatectomy versus transarterial chemoembolization for ruptured spontaneous hepatocellular carcinoma, Lee et al. (50) suggested that staged hepatectomy may provide better long-term survival than TACE alone. Moreover, the 1-, 3-, and 5-year OS of patients who underwent staged hepatectomy were 88.7%, 58.4%, and 43.8%, respectively, which is a relatively good result, and the patients do not have a very poor prognosis like T4 liver cancer.

Since the patients’ vital signs are much more stable than during emergency surgery, this pattern of staging surgery has become the current consensus. Even its prognosis is comparable to the prognosis of some non-ruptured patients (13, 62, 76).

For prognostic factors affecting the surgical treatment of ruptured hepatocellular carcinoma. In Wei Zhang et al’s study (53), In TACE and Liver resection (LR) group, tumor size ≥ 10 cm and tumor non-capsule were independent risk factors for poor prognosis of OS and RFS. In the TACE group, AFP ≥ 1000 ng/ml was also a risk factor for prognosis. Hypovolemic shock was also a risk factor for OS in the LR group. In Xiang-Jun Han et al’s study (77), they concluded that antineoplastic therapy, tumor length, tumor number, and BCLC stages were the most critical predictors of OS. At the same time, pathological factors play an important role in the postoperative prognosis of HCC patients, which is often reported in many pieces of literature. An ongoing study by our team combines multiple pathological factors to form a pathological score to predict the postoperative prognosis of rHCC and HCC patients.

### (4) Microwave Coagulation and Radiofrequency Ablation

Microwave coagulation can coagulate the tumor tissue near the bleeding point and block the bleeding blood vessels to achieve the hemostatic effect. At the same time, it also provides some oncological benefits by targeting local tumors and inactivating some cancer cells, and it can reduce the risk of peritoneal carcinomatosis (78, 79). It is an effective method for hemostasis, but the recurrence rate of the tumor and the thoroughness of hemostasis are still worthy of further exploration and study. Maria Baimas-George et al. (80) demonstrated that using laparoscopic MWA irrigation might provide advantages in treating ruptured HCC by performing posterior TAE or TACE followed by laparoscopic Microwave coagulation irrigation in 10 of 15 patients.

Radiofrequency ablation involves the insertion of a radiofrequency needle into the tumor under the guidance of ultrasound; the thermal effect of radiofrequency forms a reaction band around the tumor, reducing the tumor’s blood supply, leading to hemostasis. No institutions have reported the use of RFA to treat tumor rupture.

Therefore, microwave coagulation and radiofrequency ablation are less frequently used to treat HCC rupture at present. Radiofrequency ablation is more used for recurrence after hepatectomy (78, 79, 81–83).

## Conclusion

Spontaneous rupture and hemorrhage of HCC is believed to be one of the fatal emergencies in clinical practice (3, 13, 56). In the current HCC staging system, tumor rupture is arbitrarily classified as a T4 disease (11), However, in practical clinical work, some patients with rHCC can also achieve outcomes through effective treatment, and the prognosis is not as poor as patients with non-ruptured T4 disease. In terms of treatment for rHCC, Liver tumor is removed for patients with stable vital signs and Child grades A and B. Patients with shock and poor liver function are treated with anti-shock therapy, and hepatic artery embolization, radiofrequency ablation, and microwave coagulation to stop bleeding. When the patient’s condition improves and the liver function is tolerable, staged hepatectomy is performed and it is currently considered to be one of the best treatment modalities. With aggressive treatment, rHCC patients still have a favorable prognosis (50, 84).

## Author Contributions

FX wrote the paper. PZ and MZ provided the idea. BZ, PZ, and XC reviewed and EN edited the manuscript. All authors read and approved the manuscript.

## Funding

The research is funded by (1) Natural Science Foundation of Hubei Province (2019CFB433); (2) Hengrui Hepatobiliary and Pancreatic Malignant Tumor Research Fund-Youth Research Fund (CXPJJH11800001-2018306); (3) Sources of funding: Key project of science and technology in Hubei Province (2018ACA137); (4) General Project of Health Commission of Hubei Province (WJ2021M108).

## Conflict of Interest

The authors declare that the research was conducted in the absence of any commercial or financial relationships that could be construed as a potential conflict of interest.

## Publisher’s Note

All claims expressed in this article are solely those of the authors and do not necessarily represent those of their affiliated organizations, or those of the publisher, the editors and the reviewers. Any product that may be evaluated in this article, or claim that may be made by its manufacturer, is not guaranteed or endorsed by the publisher.

## Abbreviations

HCC, hepatocellular carcinoma; rHCC, ruptured hepatocellular carcinoma; US, ultrasound; CT, computed tomography; VEGF, vascular endothelial growth factor; AFP, Alpha-fetoprotein; TAE/TACE, transcatheter arterial embolization/chemoembolization; OS, overall survival; RFS, recurrence-free survival.
